# Prediction of Fishman’s skeletal maturity indicators using artificial intelligence

**DOI:** 10.1038/s41598-023-33058-6

**Published:** 2023-04-11

**Authors:** Harim Kim, Cheol-Soon Kim, Ji-Min Lee, Jae Joon Lee, Jiyeon Lee, Jung-Suk Kim, Sung-Hwan Choi

**Affiliations:** 1 Department of Orthodontics, Institute of Craniofacial Deformity, Yonsei University College of Dentistry, Seoul, Republic of Korea; 2 Private Clinic, Seongnam-si, Republic of Korea; 3 Crescom, Seongnam-si, Republic of Korea

**Keywords:** Orthodontics, Paediatric dentistry

## Abstract

The present study aimed to evaluate the performance of automated skeletal maturation assessment system for Fishman’s skeletal maturity indicators (SMI) for the use in dental fields. Skeletal maturity is particularly important in orthodontics for the determination of treatment timing and method. SMI is widely used for this purpose, as it is less time-consuming and practical in clinical use compared to other methods. Thus, the existing automated skeletal age assessment system based on Greulich and Pyle and Tanner-Whitehouse3 methods was further developed to include SMI using artificial intelligence. This hybrid SMI-modified system consists of three major steps: (1) automated detection of region of interest; (2) automated evaluation of skeletal maturity of each region; and (3) SMI stage mapping. The primary validation was carried out using a dataset of 2593 hand-wrist radiographs, and the SMI mapping algorithm was adjusted accordingly. The performance of the final system was evaluated on a test dataset of 711 hand-wrist radiographs from a different institution. The system achieved a prediction accuracy of 0.772 and mean absolute error and root mean square error of 0.27 and 0.604, respectively, indicating a clinically reliable performance. Thus, it can be used to improve clinical efficiency and reproducibility of SMI prediction.

## Introduction

The assessment of skeletal maturation is essential not only in medical or forensic, but also in dental fields, especially in orthodontics and pediatric dentistry. In growing patients, skeletal maturity is an important factor to consider for optimal timing and success of orthodontic treatment. Especially in patients with skeletal discrepancies between the upper and lower jaws who require growth modification, the timing of orthodontic treatment is essential for the treatment outcomes, as the redirection of jaw growth is effective only during certain stages of growth^1^. Therefore, the evaluation of skeletal maturation is indispensable for orthodontic diagnostics and treatment planning, as well as for the evaluation of treatment progress and prognosis.

It has been reported in previous studies that maturational development shows large individual variations, leading to discrepancies between the chronological and skeletal age^2,3^. Hand-wrist radiographs have been widely utilized for the evaluation of skeletal maturation, given the relatively low dose of radiation exposure and quick and uncomplicated and non-invasive procedure of x-ray taking. It is based on the principle that the osseous development in the hand and wrist areas is closely related with general maturational development^4^. The recognition of osseous features in the hand-wrist areas characteristic of each maturation level underlies the estimation of skeletal age. Different methods have been proposed for the analysis of hand-wrist radiographs.

As one of the most widely used methods, Greulich and Pyle (GP) is based on an atlas consisting of standard hand-wrist radiographs for each sex and skeletal age. The reference images were obtained from middle-classed Caucasian children between 1931 and 1942 in Ohio, United States^5^. While this atlas-based comparative method is convenient for clinical use, it assumes that the sequence and pattern of ossification and the corresponding maturation level are identical in all individuals^6^. On the other hand, Tanner-Whitehouse (TW) method is a bone-specific technique, which evaluates and scores multiple skeletal maturity indicators and determines the skeletal age according to the total score, referred to as skeletal maturity score (SMS). There are several variations of TW, which differ in the number of evaluated sites and the relationship between SMS and the corresponding skeletal age. TW3 was published in 2001 to reflect the secular trend of maturational development^7^. While TW3 is less sensitive to individual variations compared with GP, its application in everyday clinical practice is rather difficult, because the evaluation of multiple maturity indicators is complicated and time-consuming.

Skeletal maturity indicators (SMI) introduced by Fishman, an American orthodontist, in 1981 are widely used for the assessment of skeletal maturity in orthodontic patients. In contrast to the forensic or medical fields, the determination of skeletal age in number of years, or the estimation of final adult height is not of great importance in orthodontics. The main focus is rather on the timing of pubertal growth spurt and growth completion, as the course of orthodontic treatment may vary depending on these factors. Therefore, the analytic methods proposed for the application in the orthodontic field, such as Fishman’s SMI^4^ and Hägg and Taranger method^8^ are simplified to focus on the key stages of maturational development, which are relevant for decision makings during orthodontic treatment^9^.

With the advances in artificial intelligence (AI), numerous studies have been reported concerning the use of deep learning and neural networks for the evaluation and analysis of radiographs in various medical and dental fields. While the interest in the automation for clinical efficiency is ever increasing, there are also concerns about the accuracy and clinical validity of such automated systems^10^. The present study aimed to evaluate the performance of an AI-based automated assessment system for SMI prediction.

## Materials and methods

### Model development

**Figure 1:**
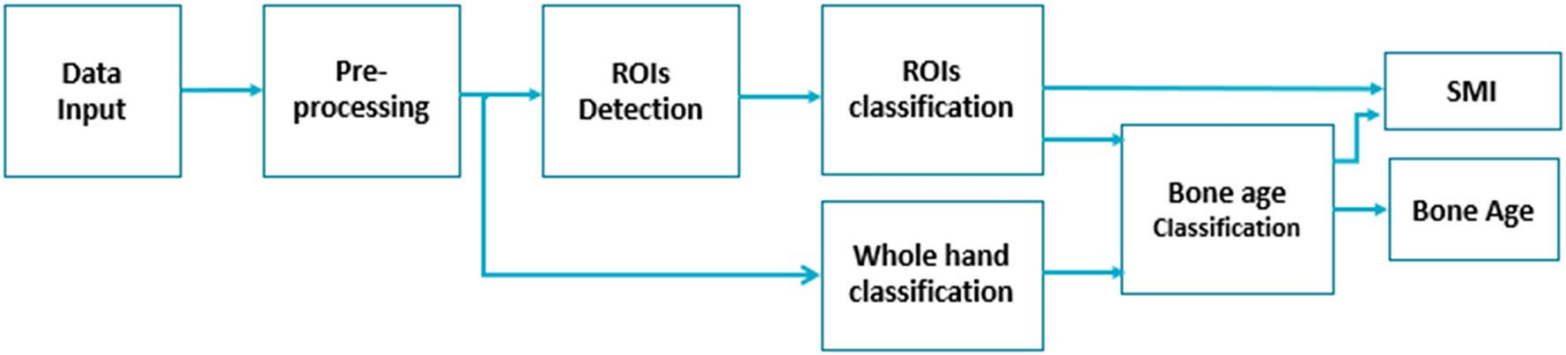
Overview of the steps involved in the modified SMI-based skeletal maturation assessment system. In the pre-processing step, the image size of hand-wrist radiographs is adjusted, and right-hand radiographs are mirrored. In addition to the detection and classification of each ROI, the evaluation of the whole hand as a holistic image is performed to enhance the performance by including maturity features that are not parts of the ROIs.

The existing GP and TW3-based bone age assessment system (MediAI-BA, Crescom Inc., Korea)^11^ was further trained and modified to include the evaluation of SMI. The pre-trained system employed a hybrid approach combining the advantages of TW3 and GP methods to improve the performance of skeletal age prediction^11,12^. The automated assessment system for SMI was developed based on the same principle applied to the GP and TW3-based system, consisting of three major steps: (1) automated detection of region of interest (ROI); (2) automated evaluation of skeletal maturity of each region; and (3) SMI stage mapping (Fig. 1). In this version, the detection algorithm for each ROI was developed using a detection Transformer model^13^, which utilizes learnable queries to search image features from the output of Transformer encoders and bipartite graph matching for detection. For automated evaluation of skeletal maturity of each region, the classification algorithm was developed using Swin Transformer model^14^, which is a hierarchical vision transformer deep learning model with a window-shifting method. Data augmentation techniques, such as random resize, random crop, brightness adjustment, contrast adjustment, CLAHE, and random noise, were applied during deep learning training.

**Figure 2:**
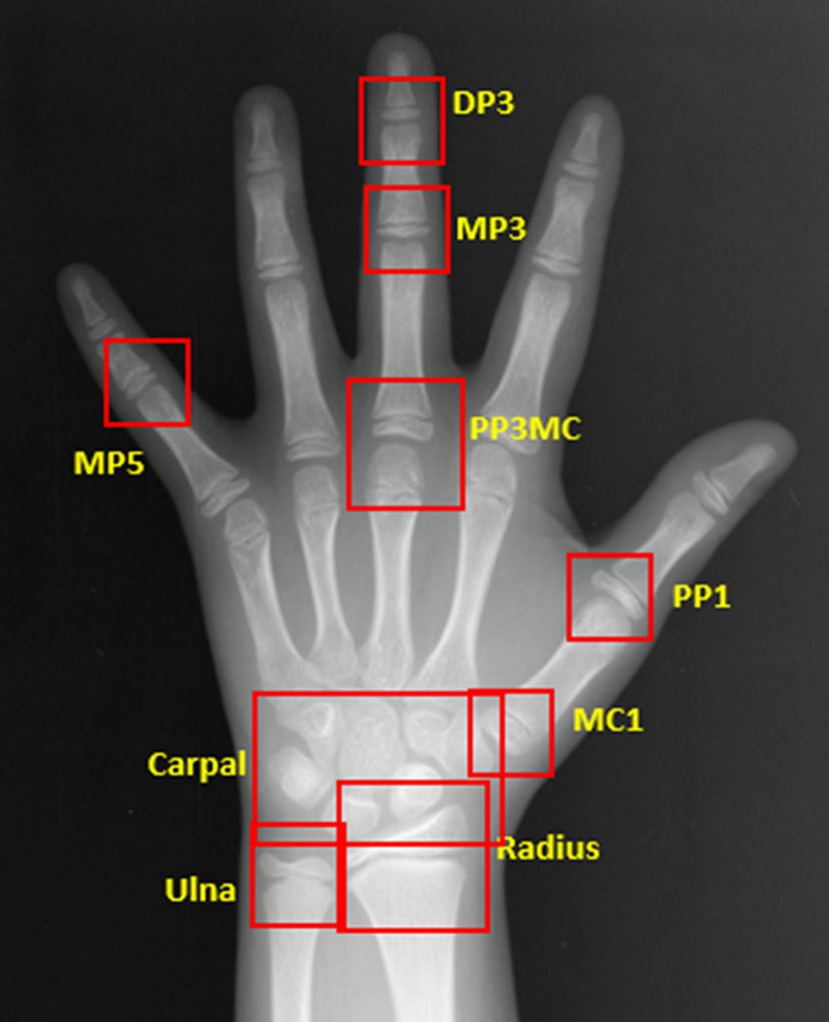
Automated detection of regions of interest. In addition to the ROIs used in the TW3 method, all of the six ROIs used for Fishman’s SMI, as well as the carpal bones are detected. (DP3, 3rd distal phalanx; MP3, 3rd middle phalanx; MP5, 5th middle phalanx; PP3MC, 3rd proximal phalanx/metacarpal; PP1, 1st proximal phalanx, MC1, 1st metarcarpal).

**Figure 3:**
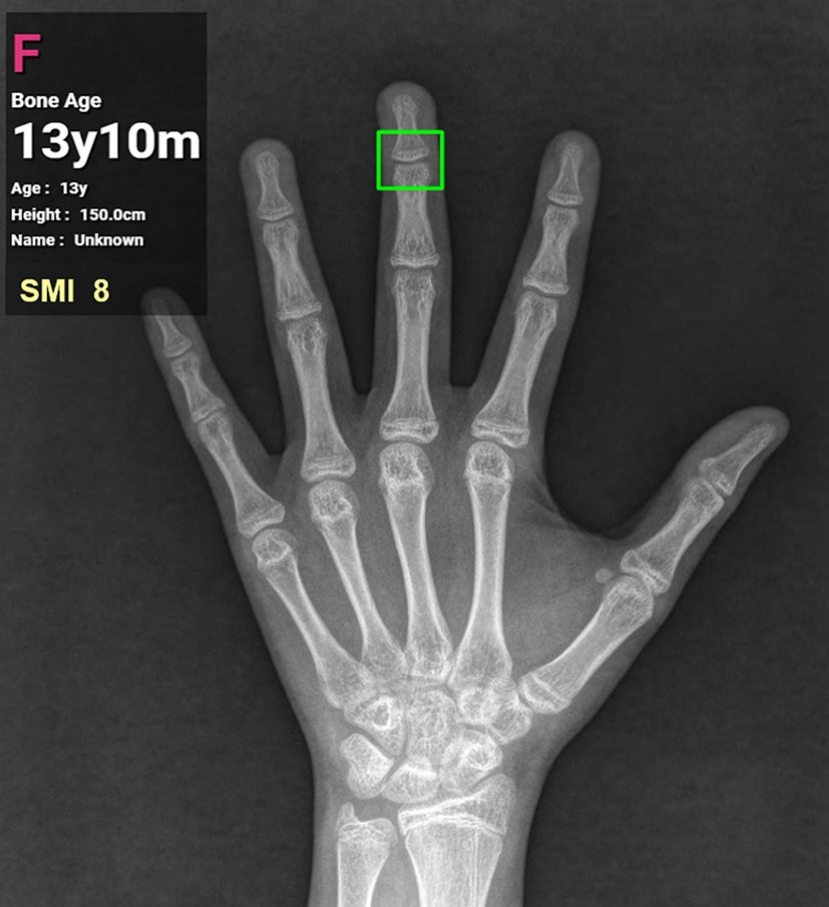
Result screen displaying the SMI stage and bone age predicted by the SMI-modified automated skeletal maturation assessment system.

In addition to the major areas of growth plate evaluated for the TW3 method and the carpal region, all regions subject to the evaluation of SMI were included as ROIs (Fig. 2). In particular, MP5 (fifth middle phalanx) and PP1 (first proximal phalanx) were added by utilizing the detection algorithm based on the work by Li et al.^13^ RetinaNet, a deep convolutional neural network (CNN), was utilized for the automated detection of ROIs. Subsequently, each of the detected regions was analyzed for skeletal maturity level based on the morphological changes, such as epiphyseal widening, presence of sesamoid, epiphyseal capping and epiphyseal fusion. For image classification a vision transformer-based deep learning model was utilized. The final skeletal age was calculated by analyzing and integrating the probability of skeletal maturity of each area. Irrespective of the skeletal age prediction, skeletal maturity level of each of the six regions relevant for SMI evaluation was analyzed for SMI stage mapping. The final SMI stage was calculated by integrating the skeletal maturity and age of each ROI, and the final skeletal age. Both SMI and skeletal age were outputted as results (Fig. 3).

The primary verification of this SMI-modified system was carried out using the verification dataset, and the system underwent further modifications and adjustments according to the results. Subsequently, the accuracy of the finally modified system was tested using a new set of hand-wrist radiographs from a different institution, which had not been introduced to the system previously.

### Subjects

The dataset used for the primary verification consisted of hand-wrist radiographs obtained from 2593 growing patients as part of orthodontic diagnosis between April 2019 and July 2022 at a private orthodontic clinic in Korea. The accuracy of the final SMI-modified system was tested using hand-wrist radiographs of 711 patients, who visited the Department of Orthodontics, Yonsei University Dental Hospital in Seoul, Korea between August 2019 and February 2021. The exclusion criteria for both of the datasets were as follows: (1) congenital craniofacial anomalies or syndromes affecting growth, (2) history of growth hormone treatment, (3) history of systemic diseases and medication, and (4) insufficient quality of radiographs affecting the evaluation of SMI.

The study protocol was approved by the Institutional Review Board of Yonsei Dental Hospital (IRB No. 2-2022-0030), and adhered to the Declaration of Helsinki (2013). The requirement of informed consent and ethical approval was waived in view of the retrospective and non-interventional design of the study. All procedures were performed irrespective of the study as part of the routine care.

### Evaluation of SMI

SMI staging performed by the observers strictly followed the descriptions provided in the original article by Fishman. Eleven different skeletal maturity indicators, which are numbered through SMI 1 to 11, were recognized by assessing six sites of the hand and wrist areas^4^. In addition, SMI 0 was included to express the maturation level earlier than that of SMI 1^15,16^. For each dataset, the evaluation of SMI was undertaken by three different orthodontists, who have multiple years of clinical experience in orthodontics. The ground-truth SMI was determined as the stage that was the most frequently chosen by the observers. If no agreement could be reached, a fourth observer was asked for additional evaluation. To minimize any bias, the observers were unaware of the chronological age of the patients and the SMI stage determined by other observers at the time of evaluation.

### Statistical analysis

For statistical analysis and data visualization, Microsoft Excel for Windows (version 2019; Microsoft Corp., WA, USA) and Scikit-learn library in Python were used. The performance of the automated SMI assessment system was evaluated by computing mean absolute error (MAE) and root mean square error (RMSE) using the following formulas.

1 $${\text{MAE}} = \frac{1}{n}\mathop \sum \limits_{i = 1}^{n} \left| {yi {-} xi} \right|$$

2 $${\text{RMSE}} = \sqrt {\frac{1}{n}\mathop \sum \limits_{i = 1}^{n} \left( {yi {-} xi} \right)^{2} }$$

In both formulas, $n$ is the number of evaluated hand-wrist radiographs, and $yi$ and $xi$ represent the ground-truth and AI-predicted SMI, respectively. MAE and RMSE were calculated for the overall performance of the system, as well as for each of the SMI stages.

Furthermore, confusion matrix was generated for visual presentation of the correctly and incorrectly predicted SMI. The overall prediction accuracy was calculated by dividing the number of correct predictions by the total number predictions. In addition, sensitivity and specificity of each SMI stage, as well as the overall sensitivity and specificity were obtained.

## Results

**Figure 4:**
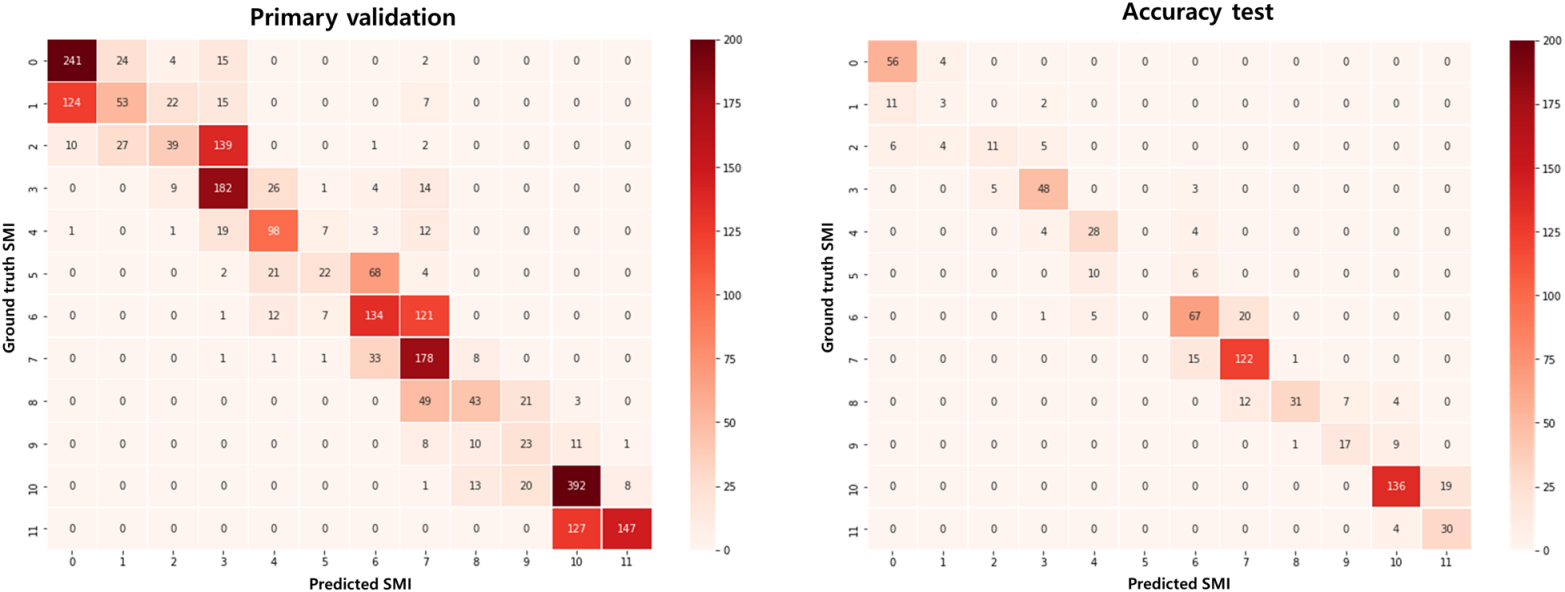
Confusion matrices showing the results of primary validation and accuracy test following fine tuning.

The overall prediction accuracy obtained following the primary validation of the automated SMI assessment system was 0.599 with MAE of 0.499. The confusion matrix exhibited a diagonal pattern, which implies an overall correct prediction of SMI (Fig. 4). However, the AI-predicted SMI 7 showed a large range of incorrect predictions. According to the results of the primary validation, the algorithm of SMI mapping underwent further adjustments to improve the performance of the system.

Upon adjusting the algorithm, the performance of the final model was evaluated using the test dataset consisting of hand-wrist radiographs of 711 patients (361 males and 350 females). The patient age at the obtainment of radiograph ranged from 6 to 18 years, with the mean age of 11.93 ± 2.43 years. As can be seen in the confusion matrix, most of the predictions remained in the diagonal, with only a few outliers (Fig. 4). The overall prediction accuracy was 0.772, indicating that the AI-predicted SMI corresponded to the ground-truth SMI in 77.2% of the cases. Considering the uneven distribution of the SMI stages in the dataset, balanced accuracy was obtained additionally. It was slightly smaller than the prediction accuracy with 0.704. When the tolerance range was expanded to within 1 SMI stage, the prediction accuracy increased to 0.963. The overall MAE and RMSE were 0.27 and 0.604, respectively. As a linear solution, MAE weighs errors equally in the average regardless of their magnitude. On the other hand, RMSE weighs large errors more heavily. Both MAE and RMSE were the smallest for SMI 0 with 0.067 and 0.258, respectively. The largest MAE was calculated for SMI 5 (1.0) followed by SMI 1 (0.938) and 2 (0.846). On the other hand, RMSE was bigger for SMI 2 (1.177) than SMI 1 (1.09) and SMI 5 (1.0). Accordingly, the highest sensitivity was found for SMI 0, and the lowest for SMI 5, followed by SMI1. The overall specificity was 0.978, with the highest values found for SMI 5 (1.0) and SMI 8 (0.997), and the lowest for SMI 7 (0.944) (Table 1).

**Table 1:** Performance of automated SMI assessment system.

SMI	Sensitivity	Specificity	MAE	RMSE
0	0.933	0.972	0.067	0.258
1	0.188	0.990	0.938	1.09
2	0.423	0.993	0.846	1.177
3	0.856	0.982	0.25	0.756
4	0.778	0.978	0.333	0.745
5	0.000	1	1	1
6	0.720	0.955	0.355	0.726
7	0.884	0.944	0.116	0.341
8	0.574	0.997	0.5	0.805
9	0.630	0.990	0.37	0.609
10	0.877	0.969	0.123	0.350
11	0.882	0.972	0.118	0.343
Overall	0.646	0.978	0.27	0.604
Prediction accuracy 0.772			Balanced accuracy 0.646	

SMI, skeletal maturity indicators; MAE, mean absolute error; RMSE, root mean square error.

## Discussion

The present study is the first to report an AI-based automated system for the assessment of Fishman’s SMI, which is widely used in dental fields, especially in orthodontics. In recent years, an increasing number of studies have been reported on the automation of skeletal maturation assessment using AI for the improvement of clinical efficiency and reproducibility. The vast majority of these proposed systems are based on TW3 method^12,17–19^, as it has several advantages over other methods. Firstly, it takes into account the variability of skeletal maturation pattern in different populations and ethnic groups. Unlike its predecessor, TW2, which derived from a sample of British children^20^, TW3 was developed using additional data from multiple ethnic groups and populations^7^. Later studies have also shown its validity in various populations, including Korean children^21–23^. Furthermore, TW3 method offers a comprehensive evaluation of skeletal maturity by assessing multiple bones, leading to a more thorough analysis of skeletal development and improved reliability. On the other hand, a major drawback of TW3 is the complexity and time required to obtain results. This limitation has been addressed through the use of AI-based systems. Another limitation of TW3 is that it does not account for changes in the trend of maturational development over time. This could lead to potential obsolescence of the method, much like its predecessor TW2. Therefore, periodic updates are necessary to maintain the validity and reliability of the TW method^7^.

Similarly, the GP method is susceptible to changes in the trends of maturational development, as well as to variations resulting from differences in ethnicity, regional factors, and environmental influences^24–26^. Unlike the Tanner-Whitehouse method, which has undergone revisions since its inception, the GP method has not been updated since its introduction in 1959^5^. In other words, it is solely based on the initial reference hand-wrist radiographs of Caucasian children obtained over 80 years ago. According to the study by Mansouvar et al., the GP method is reliable for Caucasian and Hispanic children, but not for African/American and Asian groups^27^. A study with the sample of Korean population also concluded that the rates of skeletal development provided by GP is not applicable to Korean children^28^.

In contrast to TW3 and GP, Fishman's SMI provides a staging system for assessing maturation levels, which is not reliant on skeletal age^4^. Consequently, SMI is not subject to fluctuations in the trend of maturational development and differences arising from factors such as ethnicity. In simpler terms, SMI allows for an intuitive determination of an individual's skeletal maturity level, without necessitating consideration of additional factors. When assessing skeletal maturity for orthodontic purposes, the level of maturation in relation to chronological age is of little importance. As a result, obtaining skeletal age is not typically necessary, in contrast to its importance in medical or forensic fields. Nevertheless, SMI, like other methods, has its limitations. Since a single skeletal indicator is assigned for each stage, variations in the appearance sequence of skeletal maturity indicators or unclear indications of these indicators can lead to misstaging.

Clinicians often encounter hand-wrist radiographs with osseous maturational characteristics that are ambiguous to be classified as a certain SMI stage^29^. In case of individual variations in the sequence of skeletal maturation that do not comply with the descriptions by Fishman, SMI stage may be over- or underestimated depending on the observer, and reproducibility and reliability of SMI may be affected. It has been reported in a previous study that the prediction accuracy was relatively low for SMI stages 5 and 6. Insufficient amount of data, and large inter-observer variabilities were considered as the possible reasons^15^. Similarly, in the present study, the prediction accuracy for SMI stage 5 was found to be lower compared to other SMI stages. This may be related with the fact that there was no radiograph in the dataset used for the accuracy test that was predicted as SMI 5. However, the prediction accuracy of SMI 5 was calculated to be only 0.19 also in the primary validation, which was carried out with a larger dataset. In other words, SMI stages that are more likely to deviate from the proposed sequence are more prone to higher inter-observer variability and lower prediction accuracy. The system introduced in the present study is a hybrid approach that evaluates maturational level by integrating the GP, TW3, and SMI methods. This approach compensates for the known limitations of each system and enhances the accuracy and reproducibility of SMI predictions.

Recent advances in technology have led a notable surge in the integration of AI into dental practice for tasks such as diagnosis, radiographic analysis and treatment planning^30^. Consequently, there have been attempts to streamline and accelerate the process of skeletal maturity assessment through the application of AI^31^, as the manual assessment has been subject to criticism for its tediousness and intra- and interobserver variabilities^32,33^. Previous studies have demonstrated clinically reliable performance of deep learning-based automated systems for assessing skeletal maturity^11,17,18,34–36^. However, the majority of introduced models are based on TW3 or GP methods. Few automated systems have been proposed for the assessment of skeletal maturity using SMI. However, previous studies that investigated SMI in relation to AI focused rather on the prediction of SMI using the radiographic images of cervical vertebrae^15,37^.

The performance of various automated skeletal maturation assessment systems has been evaluated in previous studies. According to these studies, the AI-predicted skeletal age was not significantly different from the skeletal age assessed by experts^11,19^. The range of MAE reported in the literature varies from 0.39 to 2.41 years depending on the study^17,34–36,38–42^. It is notable that the models proposed during the last decade^17,34,35,38,42^ show better performance with smaller MAE compared with the models introduced earlier^36,39–41^. Since the MAE computed in the present study does not refer to skeletal age, but SMI stage, it cannot be directly compared with the results of previous studies. According to the data provided in Fishman’s study, the mean interval between SMI stages is 0.61 years for the female and 0.64 years for the male sex^4^. Based on this information, MAE of 0.27 SMI stage can be converted to approximately 0.169 years. Although this conversion may not be accurate, as it does not consider the differences in the size of the interval between the stages, the results suggest that the SMI-modified automated skeletal maturation system shows a satisfactory performance compared with previous systems.

The present study has several limitations. The size of the study population as a whole was sufficient, however, the number of observations was relatively small for some of the SMI stages. This may have affected the reliability of the accuracy measured for these subgroups. Furthermore, the data were collected retrospectively. While the data used for AI-training were collected from different institutions including different ethnicities, the datasets used for the primary validation and final evaluation consisted of hand-wrist radiographs only of a single ethnic group. Therefore, the results of the present study may not reflect possible differences between various ethnicities, or populations. In order to validate the results of the present study, and to further improve the accuracy of the proposed system, future studies with larger datasets from multiple institutions and populations are required.

## Conclusion

The hybrid SMI-reinforced automated skeletal maturation assessment system introduced in the present study was shown to deliver clinically reliable prediction of SMI with a very low prediction error. Hence, it can be efficiently utilized in dental fields to enhance clinical efficiency. It can also assist clinicians in improving the reproducibility of skeletal maturation assessment.

## Data Availability

The data underlying this article cannot be publicly shared to protect the privacy of the individuals participating in the study. The data will be shared at a reasonable request to the corresponding author.
